# The Impact of a 12-Week School-Based Mindfulness Programme on Emotional Intelligence: A Cross-National Study of Primary and Secondary Students in Spain and Italy

**DOI:** 10.3390/jintelligence14070123

**Published:** 2026-07-01

**Authors:** Ángela Lori, Eva María Padilla-Muñoz, Francisco Javier Cano-García, Antonino Raffone

**Affiliations:** 1 Ministero dell’Istruzione dell’Università e della Ricerca, 00153 Rome, Italy; a_lori8@yahoo.fr; 2 Departamento de Personalidad, Evaluación y Tratamiento Psicológicos, Universidad de Sevilla, 41018 Seville, Spain; evapadi@us.es; 3 Department of Psychology, Sapienza University of Rome, 00185 Rome, Italy; antonino.raffone@uniroma1.it; 4 School of Buddhist Studies, Philosophy & Comparative Religions, Nalanda University, Bihar 803116, India

**Keywords:** mindfulness, emotional intelligence, primary school, secondary school, cross-national study, EQ-i:YV, socio-emotional well-being, school-based intervention

## Abstract

In recent decades, the educational landscape has undergone rapid transformations, highlighting emotional intelligence (EI) and mindfulness as key constructs for students’ well-being and adaptation. This study examines the impact of a structured 12-week school-based mindfulness programme on emotional intelligence in a cross-national sample of students. The sample comprised 207 participants (52.7% from Spain and 47.3% from Italy). In Spain, the mean age was 8.65 years (SD = 0.36) in primary education and 13.71 years (SD = 0.49) in secondary education; in Italy, the mean age was 8.09 years (SD = 0.30) and 13.33 years (SD = 0.43), respectively. Using a quasi-experimental repeated-measures design, EI was assessed at pre-test, post-test, and 5-month follow-up with the EQ-i:YV and analysed using General Linear Models. In line with these objectives, results indicated no significant increase in global EI (*p* > .05), although small, domain-specific effects emerged (η^2^*p* ≈ .03–.09). Italian primary students showed improvements in General Mood and Interpersonal Competence, while Italian secondary students displayed delayed gains in Stress Management. No significant effects were observed in Spanish subsamples or in Adaptability and Intrapersonal Competence. These findings indicate modest, context-dependent effects, with limited and context-dependent educational implications.

## 1. Introduction

Growing academic pressure, emotional demands, and challenges to students’ psychological well-being have increasingly been highlighted in the educational literature (Çapar, 2025; Hawkins & Burke, 2021), reflecting the complex demands faced by students in contemporary learning environments. Within this context, Emotional Intelligence (EI) has emerged as a central construct for socio-emotional functioning and adaptive behaviour. Contemporary models of intelligence increasingly recognise that beyond cognitive ability, emotional and social competencies are essential for shaping academic success, interpersonal harmony, and long-term resilience (Bar-On, 2000, 2006; Goleman, 1995). As noted by Hawkins and Burke (2021), modern education must balance “heart, mind, and body,” cultivating wisdom and compassion alongside traditional cognitive capacities.

During childhood and adolescence—periods marked by significant neurocognitive and social development—higher levels of emotional intelligence are associated with better classroom behaviour, peer relationships, and academic engagement (Chamizo-Nieto et al., 2020; Costa & Faria, 2025; MacCann et al., 2020; Quílez-Robres et al., 2023). In addition, emotional intelligence has been linked to improved psychological adjustment and well-being, reinforcing its relevance as a key construct in educational contexts (Aroutzidis & Antonopoulou, 2025).

Parallel to the focus on emotional intelligence, mindfulness has gained prominence as a psychological training approach. Defined as present-moment awareness characterised by a non-judgmental and accepting attitude (Kabat-Zinn, 2005), mindfulness has been linked to improvements in attentional control, cognitive flexibility, and emotion regulation (Creswell, 2017; Hölzel et al., 2011; Zelazo & Lyons, 2012). From a neurocognitive perspective, these processes provide a foundation for emotion regulation and perspective-shifting (Hölzel et al., 2011). In addition, empirical studies have reported reductions in negative affect and improvements in metacognitive emotional awareness following mindfulness training (Amundsen et al., 2020; Vickery & Dorjee, 2016).

While mindfulness and emotional intelligence have often been studied disjointedly, they are theoretically convergent (Rodríguez-Ledo et al., 2018). The enhancement of attentional stability and self-awareness through mindfulness serves as a foundation for the key pillars of EI: self-management, social awareness, and relationship management (Goleman, 1998; Mettler et al., 2023). Evidence suggests that mindfulness practice fosters advances in empathy and relationship quality (Lin et al., 2025; Wong, 2019), producing reliable effects on socio-emotional outcomes in non-clinical youth populations (Carsley et al., 2018; Phan et al., 2022; Verhaeghen, 2023). Furthermore, during the emotional volatility of puberty, mindfulness may promote cognitive control and stress resilience (Monsillion et al., 2023). Recent research has also highlighted the role of mindfulness-based interventions in promoting emotional development and self-regulation in youth populations (Dariotis et al., 2023), further supporting its relevance for the study of emotional intelligence.

Given this convergence, emotional intelligence was selected as the primary outcome variable, as it provides a multidimensional and operationalisable framework to assess changes in socio-emotional competencies potentially influenced by mindfulness training.

However, the literature also highlights the need for nuanced, large-scale investigation. While some large-scale trials, such as the MYRIAD study (Kuyken et al., 2022), have noted that universal application requires careful attention to implementation conditions, these findings primarily emphasise the importance of identifying “what works for whom.” Rather than suggesting a lack of efficacy, such results underscore the necessity of high-quality, standardised programmes that account for developmental stages and cultural contexts (Baelen et al., 2023; Roeser et al., 2023).

Previous research within the same cross-national sample as the present study found that a structured 12-week mindfulness programme improved academic performance and executive functions, with effects varying by age (Lori et al., 2025). Despite these promising findings, the existing evidence remains limited and uneven. Previous studies have reported heterogeneous effects of mindfulness-based interventions, with outcomes varying across emotional domains, developmental stages, and implementation conditions (Carsley et al., 2018; Phan et al., 2022; Verhaeghen, 2023). Moreover, relatively few studies have examined how mindfulness training influences the multidimensional structure of emotional intelligence using validated instruments, particularly within cross-cultural contexts (Rodríguez-Ledo et al., 2018; van de Weijer-Bergsma et al., 2014). As a result, it remains unclear not only whether such interventions are effective, but also under what conditions and for whom they may be most beneficial. By adopting a cross-national and developmental perspective, this study aims to explore whether the effects of mindfulness training vary as a function of cultural context and educational stage.

The present study constitutes a complementary analysis within a broader research project forming part of the doctoral thesis of the first author. While a previous publication based on the same cross-national sample examined the effects of the intervention on academic performance and executive functions (Lori et al., 2025), the current study focuses specifically on emotional intelligence as a distinct outcome.

Accordingly, the objectives of the study were to: (a) examine changes in the different dimensions of emotional intelligence following participation in the programme; and (b) assess whether these changes were maintained at a 5-month follow-up. In line with these objectives, we hypothesised that: (a) mindfulness training would be associated with measurable changes in specific dimensions of emotional intelligence; (b) any observed changes might be maintained over time; and (c) developmental stage and national context would act as potential moderators of intervention effects.

## 2. Materials and Methods

### 2.1. Participants

The present study forms part of a broader research project examining the effects of a structured school-based mindfulness programme implemented in Spain and Italy. Findings concerning academic achievement and executive functions derived from the same sample have been reported elsewhere (Lori et al., 2025). The current manuscript focuses specifically on emotional intelligence outcomes.

A priori power analysis was conducted using G*Power software (version 3.1.9.7; Heinrich-Heine-Universität Düsseldorf, Düsseldorf, Germany; Faul et al., 2007) assuming a small effect size (f = 0.10), α = 0.05, power = 0.80, two groups, three repeated measurements, a correlation of 0.50 among repeated measures, and a nonsphericity correction of 1. The required minimum sample size was 164 participants.

The final sample comprised 207 students (111 boys, 96 girls) enrolled in public schools in Spain and Italy. The primary school subsample included 102 third-grade pupils (aged 8–9 years), recruited from one school in Seville (*n* = 53) and one in Florence (*n* = 49). The secondary school subsample consisted of 105 students in the first and second years of high school (aged 13–14 years). In Spain, primary school students had a mean age of 8.65 years (SD = 0.36), while secondary school students had a mean age of 13.71 years (SD = 0.49). In Italy, the mean age was 8.09 years (SD = 0.30) for primary education and 13.33 years (SD = 0.43) for secondary education. Regarding gender distribution, the Spanish subsample comprised 60 boys (55.0%) and 49 girls (45.0%), while the Italian subsample included 51 boys (52.0%) and 47 girls (48.0%). A detailed description of participant characteristics has been provided in a previous publication based on the same research project (Lori et al., 2025).

Participants were allocated to intervention and control groups based on pre-existing classroom groups within each school. Group selection was primarily based on availability and organisational criteria. Half of the classroom groups were selected through non-systematic procedures or based on specific educational needs (e.g., behavioural or academic difficulties), as determined by school staff, while the remaining groups were selected randomly. These conditions reflect the practical constraints inherent to implementing interventions in naturalistic school settings. Baseline equivalence between groups was examined, and gender was included as an interaction term in the analyses to control for potential selection biases.

Inclusion criteria were enrolment in the selected classes, parental informed consent, and student assent. Students were excluded if they presented a diagnosed physical or mental illness or if consent was not granted. The study complied with the Declaration of Helsinki and was approved by the Ethics Committee of the PhD Programme at the University of Seville (10 July 2018).

To ensure confidentiality, all data were anonymised during questionnaire administration using a self-generated code based on participants’ initials and age, which was used consistently across all assessment time points, and no personally identifiable information was included in the datasets.

No significant gender differences were found across country or educational stage. A slight imbalance between intervention and control groups was detected; therefore, gender was included as an interaction term in supplementary analyses to control for potential confounding effects.

### 2.2. Measures

Emotional intelligence was assessed using the Emotional Quotient Inventory: Youth Version (EQ-i:YV; Bar-On & Parker, 2000), a 60-item self-report instrument grounded in Bar-On’s model of social–emotional intelligence. This model conceptualises emotional intelligence as a multidimensional construct comprising emotional and social competencies that influence adaptive functioning.

The EQ-i:YV yields five composite scales: Intrapersonal Competence, Interpersonal Competence, Stress Management, Adaptability, and General Mood. A Total Emotional Quotient score is derived from the first four scales. The instrument also includes control indices (Positive Impression and Inconsistency) to assess response validity.

The questionnaire uses a four-point Likert scale ranging from 1 (“very seldom true of me”) to 4 (“very often true of me”). The instrument has been validated in Spanish (Ferrándiz et al., 2012) and Italian (Cianchetti, 2012), making it suitable for cross-national comparison.

In the present study, the EQ-i:YV showed good overall internal consistency across time (α = 0.894 pre-test, α = 0.881 post-test, and α = 0.902 follow-up). Subscale reliability coefficients were as follows: Intrapersonal (α = 0.438, 0.377, 0.453), Interpersonal (α = 0.743, 0.812, 0.811), Stress Management (α = 0.615, 0.520, 0.608), Adaptability (α = 0.869, 0.863, 0.882), and General Mood (α = 0.838, 0.805, 0.877) at pre-test, post-test, and follow-up, respectively.

### 2.3. Intervention Programme

Students in the intervention condition participated in a structured 12-week school-based mindfulness programme adapted from the Mindful Schools curriculum (Mindful Schools, 2017). A detailed description of the intervention protocol has been provided in a previous publication based on the same research project (Lori et al., 2025). Briefly, the programme consisted of weekly sessions of approximately 50–55 min, combining attentional training, breathing exercises, body awareness practices, emotional awareness activities, and prosocial components such as kindness and gratitude exercises. The programme was developmentally adapted to the students’ educational stage. While the core structure of the intervention was standardised across countries, minor adjustments were made to ensure age-appropriateness and feasibility within each educational context, without introducing substantial cultural modifications.

### 2.4. Procedure

Following certified training in the Mindful Schools curriculum, the first author delivered the intervention in both countries, ensuring consistency in the content, structure, and delivery of the programme across all groups. Participants in the control groups were informed that they were taking part in a study on mindfulness and emotional intelligence and were asked to complete the assessment questionnaires at the different time points, without receiving any intervention during the study period. The mindfulness programme was delivered during regular school hours, replacing non-core curricular content selected by each school. While students in the intervention group participated in the programme, those in the control group continued attending their regular classes and followed the standard curriculum during the same time periods. Baseline assessments (pre-test) were administered prior to the beginning of the programme. Post-test measures were collected immediately after the 12-week intervention, and follow-up assessments were conducted approximately five months later. Parents were informed that they could request feedback on their children’s results from the principal investigator upon demand.

The intervention was implemented during the third academic term in Spain and the first academic term in Italy to ensure feasibility of follow-up data collection. Assessment procedures were identical across countries. This time interval was selected to assess the maintenance of intervention effects over a medium-term interval while aligning with the academic calendar, ensuring that data collection was completed before the end of the school year and minimising participant attrition. Implementation conditions varied across school contexts due to organisational factors such as scheduling constraints, teacher involvement, and classroom dynamics, reflecting the naturalistic conditions of school-based interventions.

### 2.5. Design

A quasi-experimental repeated-measures design was employed, involving two non-randomised groups (intervention vs. control). Given the quasi-experimental nature of the design, causal inferences should be interpreted with appropriate caution. The independent variable was participation in the mindfulness programme (yes/no). Country (Spain vs. Italy) and educational stage (primary vs. secondary) were examined as between-subject factors. Emotional intelligence scores obtained at three time points (pre-test, post-test, follow-up) served as within-subject variables.

### 2.6. Data Analysis

Statistical analyses were conducted using IBM SPSS Statistics (Version 29). Descriptive statistics were calculated for all variables. Internal consistency of the EQ-i:YV scales was assessed using Cronbach’s alpha. To test the study hypotheses regarding changes in emotional intelligence over time, a General Linear Model (GLM) with repeated measures was performed. Time (pre-test, post-test, follow-up) was treated as the within-subject factor, and group (intervention vs. control), country, and educational stage were included as between-subject factors. Interaction effects between time and group were examined to determine intervention effects. Where significant higher-order interactions emerged (e.g., time × group × country), separate analyses were conducted. Statistical significance was set at *p* < .05. Effect sizes were calculated using partial eta squared (η^2^*p*) and interpreted according to established thresholds (small: 0.010–0.059; medium: 0.060–0.137; large: >0.138). Given that SPSS-derived η^2^*p* may overestimate effect sizes in repeated-measures designs, effect sizes were manually computed following Bakeman (2005). No formal corrections for multiple comparisons were applied, as the analyses were based on a predefined set of theoretically driven contrasts within the General Linear Model framework.

## 3. Results

To facilitate interpretation, analyses were conducted for the total Emotional Quotient (EQ total index) and subsequently for the individual EQ-i:YV subscales. Where relevant, interaction effects involving country and educational stage are reported.

### 3.1. Total Emotional Intelligence (EQ Total Index)

Results for the total Emotional Quotient index are presented in Table 1. No significant Time × Group interaction was observed for the overall sample, indicating that participation in the mindfulness programme was not associated with a generalised increase in global emotional intelligence across the cohort.

When analyses were disaggregated by country, a different pattern emerged. In the Italian sample, the intervention group showed a modest improvement at post-test relative to controls. However, this effect was not sustained at follow-up, suggesting that gains in global emotional intelligence were short-term and context-specific.

**Figure 1:**
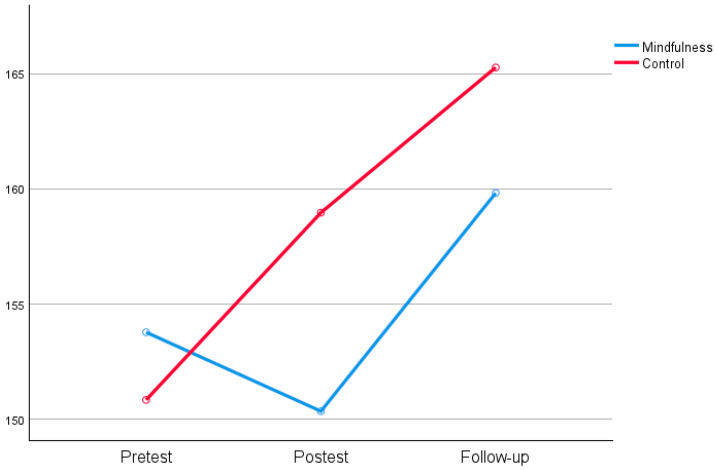
Estimated Marginal Means of Total Emotional Quotient (EQ Total Index) Across Time in Italian Primary School Students by Group.

Further differentiation by educational stage revealed that the most pronounced effect occurred among Italian primary school students, who displayed a clear post-intervention increase compared to their control counterparts. Although this improvement attenuated at follow-up, the pattern indicates greater responsiveness to the intervention among younger students within this national context (see Figure 1). In contrast, no reliable intervention-related changes were observed in the Spanish subsamples, nor among secondary school students overall.

Taken together, these findings suggest that the impact of the mindfulness programme on global emotional intelligence was not uniform, but rather dependent on developmental stage and national context. Improvements in the total EQ index were limited in scope and primarily driven by the Italian primary school group.

### 3.2. Emotional Intelligence Subscales

When the EQ-i:YV subscales were examined separately, a differentiated and context-dependent pattern emerged. Rather than uniform improvements across domains, intervention-related changes were confined to specific emotional components and particular subsamples. The most consistent effects were observed in General Mood and Interpersonal Competence, with a delayed and context-specific effect emerging in Stress Management, whereas Intrapersonal Competence and Adaptability remained largely unchanged.

#### 3.2.1. Stress Management Subscale

As shown in Table 2, no overall intervention effect was observed for the total sample in Stress Management. At the aggregate level, intervention and control groups followed comparable trajectories across assessment points. Most subsamples similarly showed no reliable post-intervention differentiation.

**Figure 2:**
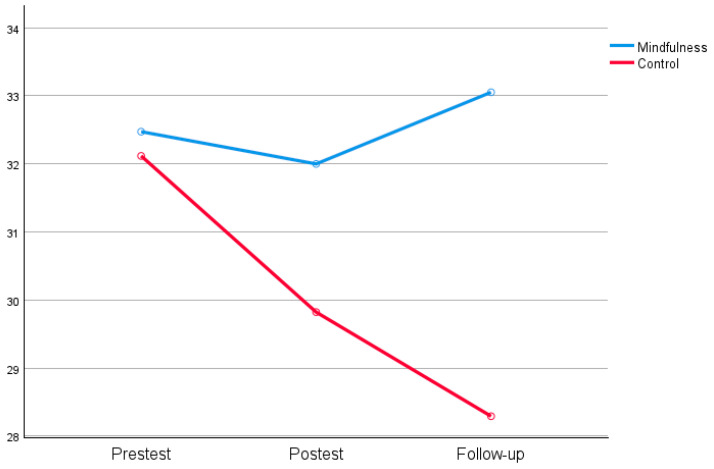
Estimated marginal means of stress management subscale across time in Italian secondary school students by group.

A distinct pattern emerged, however, within the Italian secondary school subsample. In this group, an improvement became evident at follow-up rather than immediately after the intervention, suggesting that gains in stress regulation may have consolidated over time (see Figure 2). This delayed effect was not observed among Spanish secondary students or within primary school groups.

Overall, changes in Stress Management appear developmentally and contextually contingent, with the most notable improvement observed among Italian adolescents at follow-up.

#### 3.2.2. General Mood Subscale

As presented in Table 3, no significant effect was detected for the total sample in General Mood. Nevertheless, country-level analyses revealed that improvements were concentrated within the Italian subsample.

**Figure 3:**
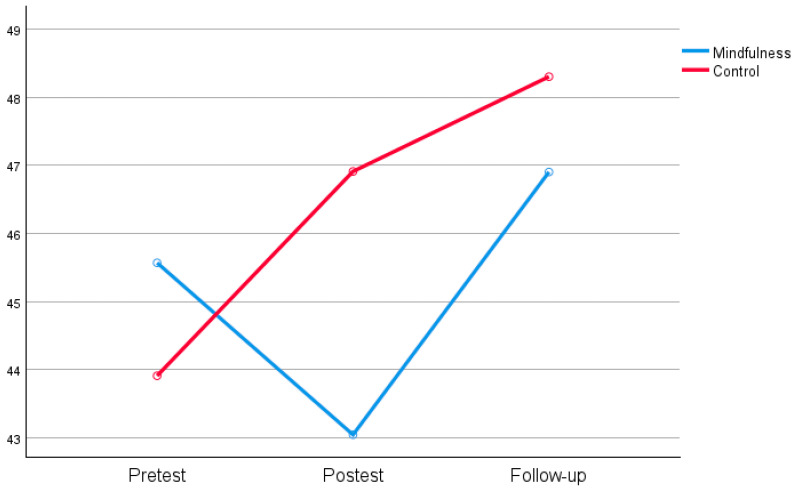
Estimated marginal means of general mood subscale across time in Italian primary school students by group.

The most pronounced change occurred among Italian primary school students at post-test, where the intervention group demonstrated a clear increase relative to controls. This effect diminished at follow-up, indicating that improvements were primarily short-term (see Figure 3). Other Italian subsamples showed smaller or less stable changes, while Spanish groups did not exhibit consistent intervention-related variation.

Thus, improvements in General Mood were largely confined to younger students within the Italian context and were strongest immediately following the intervention.

#### 3.2.3. Interpersonal Competence Subscale

Table 4 shows that Interpersonal Competence displayed modest intervention-related variation. At the whole-sample level, a short-term post-test improvement was observed, although this effect was not maintained at follow-up.

When analysed by country, clearer post-intervention differentiation emerged within the Italian sample, whereas Spanish subsamples showed no reliable changes. Some persistence of effects was observable in particular subsamples at follow-up, especially among secondary students, but these patterns were not consistent across contexts.

These findings suggest that the impact of the mindfulness programme on interpersonal functioning was limited in magnitude and unevenly distributed across national and developmental groups.

#### 3.2.4. Intrapersonal Competence Subscale

As indicated in Table 5, Intrapersonal Competence did not show meaningful intervention-related changes. Across the total sample and all subsamples, intervention and control groups displayed largely parallel trajectories from pre-test to follow-up.

Although minor fluctuations were present at certain time points, these variations were not systematically attributable to participation in the mindfulness programme. Overall, the intervention did not produce measurable changes in the intrapersonal dimension of emotional intelligence as operationalised in the EQ-i:YV.

#### 3.2.5. Adaptability Subscale

As shown in Table 6, Adaptability did not exhibit consistent intervention-related changes across the total sample or within any of the subsamples. Intervention and control groups followed comparable trajectories from pre-test to follow-up, with no systematic differentiation attributable to participation in the mindfulness programme.

Although minor fluctuations were observed at certain assessment points, these variations were not stable across time or contexts and did not indicate a clear pattern of intervention impact. Both primary and secondary school groups, as well as Spanish and Italian subsamples, demonstrated broadly parallel trends.

Overall, the findings suggest that the mindfulness intervention did not produce measurable changes in the adaptability dimension of emotional intelligence, as assessed by the EQ-i:YV.

## 4. Discussion

The present study investigated whether a structured 12-week school-based mindfulness programme could influence emotional intelligence (EI) in primary and secondary school students. While global transformations across the entire sample were not uniform, only limited and context-dependent effects were observed, with some statistically significant but generally small changes across specific emotional domains and subsamples, providing preliminary and limited support for mindfulness as a potentially beneficial targeted intervention in specific educational contexts.

A key finding of this study is the absence of significant effects in several groups, particularly within the Spanish subsamples. This lack of change suggests that the intervention, as implemented, may not have been sufficient to produce consistent improvements in emotional intelligence across contexts. These results highlight the importance of considering contextual and developmental factors when evaluating the impact of school-based mindfulness programmes.

### 4.1. Mechanisms of Emotional Regulation and Positive Change

The domains most responsive to the intervention—General Mood, Interpersonal Competence, and Stress Management—are consistent with the core processes emphasised in mindfulness training, such as non-reactivity, heightened emotional awareness, and a prosocial orientation (Hölzel et al., 2011).

When interpreted alongside earlier analyses of this cohort (Lori et al., 2025), which showed improvements in executive functions and behavioural regulation, a coherent pattern emerges. Neurocognitive models suggest that mindfulness strengthens prefrontal regulatory networks involved in attentional control and emotion modulation, thereby facilitating adaptive emotional responding (Creswell, 2017; Dunning et al., 2019). These regulatory gains likely constitute the foundational mechanisms through which mindfulness exerts its influence on emotional intelligence, fostering a targeted modulation of affective processes rather than a broad, indiscriminate transformation. Although these mechanisms have been proposed in previous research, they were not directly assessed in the present study and therefore cannot be used to explain the observed results. Consequently, these interpretations should be considered with caution.

### 4.2. Impact on Mood and Interpersonal Skills

The significant improvements in General Mood and Interpersonal Competence observed in this study are partially consistent with previous research. The positive results among primary students, for instance, reflect the findings of Amundsen et al. (2020) and Vickery and Dorjee (2016), who reported marked reductions in negative affect and strong improvements in meta-cognitive emotional awareness.

Furthermore, the gains in interpersonal skills are comparable to outcomes observed by Lin et al. (2025) and Wong (2019), where mindfulness led to significant advances in empathy and relationship quality. These findings are broadly aligned with previous research (Carsley et al., 2018; Phan et al., 2022; Verhaeghen, 2023), although the small magnitude of the effects observed in the present study suggests that their practical relevance may be limited.

### 4.3. Developmental Plasticity and Stress Resilience

The stronger effects observed among Italian primary school students may partly reflect developmental differences. During late childhood, socio-emotional competencies and executive control systems are still consolidating, making students particularly receptive to contemplative practices. Similarly, the positive impact on Stress Management among adolescents—supported by studies like Dariotis et al. (2023) and Monsillion et al. (2023)—suggests a potential association between mindfulness and cognitive control and resilience during the emotional volatility of puberty.

From a theoretical perspective, mindfulness-based programmes have been described as contributing to ‘emotionally inspired educational settings’ (Aroutzidis & Antonopoulou, 2025) and to more holistic educational approaches that integrate ‘heart, mind, and body’ (Hawkins & Burke, 2021). However, these broader educational processes were not directly assessed in the present study and, therefore, should be interpreted with caution.

Several alternative explanations should also be considered. For instance, maturation processes, classroom dynamics, or teacher expectations may have influenced the observed patterns. In addition, the naturalistic implementation of the programme across different school contexts may have introduced variability that was not fully controlled.

## 5. Limitations

Several limitations of the present study should be acknowledged.

First, the quasi-experimental design, characterised by the absence of full randomisation and the use of non-equivalent groups, limits the strength of causal inferences. Participants were allocated based on pre-existing classroom groups and, in some cases, according to pedagogical criteria. Although efforts were made to assess baseline equivalence and control for potential confounding variables, this procedure may have introduced selection biases that should be considered when interpreting the results. Therefore, the findings should be interpreted with caution, as the observed differences between groups cannot be unequivocally attributed to the intervention.

Second, the reliance on self-report measures may have introduced response biases, limiting the objectivity of the outcomes. As a result, the findings may reflect participants’ subjective perceptions rather than objective changes in emotional intelligence.

Third, the implementation of the programme was subject to contextual variability, including differences between school settings, levels of teacher involvement, and classroom dynamics, which may have influenced the consistency of the intervention effects. In addition, although the programme was delivered by the same instructor, it was not possible to systematically monitor intervention fidelity or participant engagement, including the extent of home practice or the level of engagement during sessions, which may have contributed to variability in the results.

Another limitation is that mindfulness was not directly measured. Although mindfulness-based interventions have been consistently associated with increases in mindfulness-related processes, the absence of a direct measure prevents the examination of potential mechanisms linking the intervention to changes in emotional intelligence.

Another limitation concerns the absence of corrections for multiple comparisons, which may increase the risk of Type I error; therefore, statistically significant findings, particularly those associated with small effect sizes, should be interpreted with caution.

Furthermore, a limitation of the study concerns the internal consistency of some EQ-i subscales. While the total scale and most subscales showed acceptable to good reliability, the Intrapersonal subscale presented consistently low Cronbach’s alpha values across time points, and the Stress Management subscale showed moderate reliability. These findings suggest that results related to these specific dimensions should be interpreted with caution, particularly when analysing changes at the subscale level.

In addition, due to the fact that the intervention was administered exclusively by the first author and within the time constraints of the doctoral research period, it was not feasible to offer the programme to the control groups after the completion of the study.

Likewise, although the follow-up period was longer than in many comparable studies, it may still be insufficient to capture longer-term effects of the intervention.

Finally, the cross-national design was limited to two relatively similar European contexts (Spain and Italy), which may restrict the extent to which cultural differences can be meaningfully examined. As a result, the findings should be interpreted primarily in terms of contextual and developmental variability rather than broad cross-cultural contrasts.

## 6. Future Research

Future research should aim to employ fully randomised controlled designs to strengthen causal inference, incorporate multi-method assessment strategies beyond self-report measures, and apply appropriate statistical corrections when conducting multiple comparisons. In addition, future studies should systematically monitor programme fidelity and participant engagement, including home practice, to better understand variability in outcomes. Furthermore, research should address measurement limitations by further validating subscales with lower reliability. Future studies should also incorporate direct assessments of mindfulness in order to examine its potential role as a mediating mechanism in the relationship between mindfulness-based interventions and emotional intelligence outcomes. Future work should also explore how contextual and developmental factors, such as educational stage and national setting, moderate intervention effects, as suggested by the differential patterns observed in this study. Finally, future studies should consider the feasibility of extending interventions to control groups after study completion and examine longer-term follow-up periods.

## 7. Conclusions and Educational Implications

The findings suggest that mindfulness-based interventions may be associated with changes in specific aspects of emotional intelligence; however, these effects were generally small and context-dependent, and therefore should be interpreted with caution.

From an educational perspective, these results suggest that mindfulness programmes may represent a complementary tool rather than a standalone intervention, with potential benefits depending on contextual and developmental factors.

## Figures and Tables

**Table 1:** Total Emotional Quotient (EQ Total Index).

Sample/ Subsample	Measure	N (EG)	N (CG)	Mean (EG)	Mean (CG)	SD (EG)	SD (CG)	F	*p*	η^2^*p*	Effect
Whole	Pre	82	83	153.93	155.08	21.85	21.81	--	--	--	--
	Post	82	83	152.43	156.99	20.94	20.34	2.25	.140	.005	None
	Fol	82	83	153.17	157.71	21.37	22.02	1.66	.200	.004	None
Spanish	Pre	42	43	154.62	162.56	21.64	20.88	--	--	--	--
	Post	42	43	153.45	160.58	24.28	21.10	0.06	.800	.000	None
	Fol	42	43	151.86	159.77	23.93	21.24	0.00	.990	.000	None
Italian	Pre	40	40	153.20	147.05	22.33	20.06	--	**--**	**--**	--
	Post	40	40	151.35	153.12	16.98	19.00	6.75	**.010**	**.030**	**Small**
	Fol	40	40	154.55	155.50	18.50	22.88	3.63	.060	**.020**	**Small**
Primary school	Pre	45	44	157.80	160.14	21.95	22.48	--	--	--	**--**
	Post	45	44	155.38	163.32	21.88	21.93	2.53	.120	**.012**	**Small**
	Fol	45	44	159.18	166.07	19.59	23.52	1.41	.240	.007	None
Secondary school	Pre	37	39	149.22	149.38	21.08	19.78	--	--	--	--
	Post	37	39	148.84	149.85	19.42	15.81	0.10	.760	.000	None
	Fol	37	39	145.86	148.28	21.41	15.75	0.43	.510	.002	None
Spanish Primary school	Pre	24	21	161.33	170.33	19.83	22.49	--	--	--	--
	Post	24	21	159.79	168.10	25.49	24.84	0.02	.890	.000	None
	Fol	24	21	158.63	166.95	21.10	26.31	0.02	.900	.000	None
Spanish Secondary school	Pre	18	22	145.67	155.14	21.19	16.50	--	--	--	--
	Post	18	22	145.00	153.41	20.45	13.85	0.08	.780	.000	None
	Fol	18	22	142.83	152.91	25.06	11.91	0.02	.900	.000	None
Italian Primary school	Pre	21	23	153.76	150.83	23.99	18.39	--	--	--	--
	Post	21	23	150.33	158.96	15.99	18.36	6.45	**.010**	**.070**	**Medium**
	Fol	21	23	159.81	165.26	18.20	21.22	2.92	.090	**.031**	**Small**
Italian Secondary school	Pre	19	17	152.58	141.94	20.98	21.62	--	--	--	--
	Post	19	17	152.47	145.24	18.39	17.37	0.76	.390	.006	None
	Fol	19	17	148.74	142.29	17.48	18.13	0.70	.410	**.031**	**Small**

Note. Pre = pre-test; Post = post-test; Fol = follow-up; EG = intervention group; CG = control group. Significant results are shown in bold. Effect sizes are reported as partial eta squared (η^2^*p*).

**Table 2:** Stress Management (EQ-i:YV subscale).

Sample/ Subsample	Measure	N (EG)	N (CG)	Mean (EG)	Mean (CG)	SD (EG)	SD (CG)	F	*p*	η^2^*p*	Effect
Whole	Pre	82	84	31.82	32.86	7.26	5.96				
	Post	82	84	32.06	33.07	6.60	5.95	0.00	.970	.000	None
	Fol	82	84	31.72	32.95	6.09	6.35	0.04	.840	.000	None
Spanish	Pre	42	44	31.71	33.43	6.84	6.01				
	Post	42	44	31.93	34.25	6.70	6.25	0.24	.620	.001	None
	Fol	42	44	31.79	33.84	6.59	5.32	1.00	.310	.006	None
Italian	Pre	40	40	31.93	32.22	7.73	5.92				
	Post	40	40	32.20	31.77	6.56	5.39	0.28	.600	.002	None
	Fol	40	40	32.70	31.97	6.51	6.76	0.58	.450	.004	None
Primary school	Pre	45	45	32.93	32.87	7.68	6.50				
	Post	45	45	32.82	33.84	7.33	6.23	0.64	.420	.004	None
	Fol	45	45	32.09	34.22	7.73	6.06	2.72	.100	**.016**	**Small**
Secondary school	Pre	37	39	31.46	32.22	6.56	5.36				
	Post	37	39	31.14	32.18	5.54	5.56	1.29	.210	.009	None
	Fol	37	39	31.27	31.49	4.91	5.85	2.91	.090	**.022**	**Small**
Spanish Primary school	Pre	24	22	34.25	33.45	7.03	7.06				
	Post	24	22	33.21	34.50	7.52	7.31	1.20	.230	**.014**	**Small**
	Fol	24	22	31.83	33.73	7.09	6.33	1.72	.200	.022	**Small**
Spanish Secondary school	Pre	18	22	28.33	33.41	5.09	4.91				
	Post	18	22	31.22	34.00	5.14	5.15	0.78	.380	**.010**	**Small**
	Fol	18	22	29.39	33.95	5.75	4.23	0.11	.740	.001	None
Italian Primary school	Pre	21	23	31.43	32.30	8.27	6.02				
	Post	21	23	32.38	33.22	7.26	5.08	0.00	.980	.001	None
	Fol	21	23	32.38	34.70	8.56	5.90	0.78	.380	.008	None
Italian Secondary school	Pre	19	17	32.47	32.12	7.27	5.97				
	Post	19	17	32.00	29.82	5.90	5.31	0.99	.330	**.016**	**Small**
	Fol	19	17	33.05	28.29	3.17	6.21	4.87	**.030**	**.080**	**Medium**

Note. Pre = pre-test; Post = post-test; Fol = follow-up; EG = intervention group; CG = control group. Significant results are shown in bold. Effect sizes are reported as partial eta squared (η^2^*p*).

**Table 3:** General Mood (EQ-i:YV subscale).

Sample/ Subsample	Measure	N (EG)	N (CG)	Mean (EG)	Mean (CG)	SD (EG)	SD (CG)	F	*p*	η^2^*p*	Effect
Whole	Pre	82	83	43.41	43.80	8.04	8.56				
	Post	82	83	43.32	44.17	8.13	7.86	0.27	.600	.000	None
	Fol	82	83	43.79	44.64	8.76	8.67	0.22	.640	.000	None
Spanish	Pre	42	43	43.21	45.33	8.13	9.01				
	Post	42	43	43.76	43.63	8.10	8.35	2.88	.090	**.013**	**Small**
	Fol	42	43	43.90	44.26	8.65	9.08	1.59	.210	.008	None
Italian	Pre	40	40	43.63	42.15	8.04	7.83				
	Post	40	40	42.85	44.75	8.26	7.37	8.57	**.001**	**.034**	**Small**
	Fol	40	40	43.68	45.05	8.98	8.32	4.55	**.050**	**.022**	**Small**
Primary school	Pre	45	44	46.13	46.50	7.30	8.56				
	Post	45	44	45.11	47.55	8.09	6.91	2.42	.120	**.013**	**Small**
	Fol	45	44	47.58	48.98	6.54	7.60	0.12	.730	.006	None
Secondary school	Pre	37	39	41.11	44.74	7.74	7.57				
	Post	37	39	41.14	44.36	7.75	7.18	1.37	.240	.006	None
	Fol	37	39	39.19	44.41	8.98	7.91	0.15	.700	.009	None
Spanish Primary school	Pre	24	21	46.63	49.33	7.42	8.33				
	Post	24	21	46.92	48.24	7.70	6.77	0.50	.490	.006	None
	Fol	24	21	48.17	48.48	5.82	7.85	1.70	.190	**.019**	**Small**
Spanish Secondary school	Pre	18	22	38.67	41.50	6.83	8.06				
	Post	18	22	39.56	39.23	6.72	7.37	3.13	.080	**.032**	**Small**
	Fol	18	22	38.22	44.23	8.66	8.44	0.14	.710	.002	None
Italian Primary school	Pre	21	23	45.57	43.91	7.30	8.10				
	Post	21	23	43.05	46.91	8.22	7.12	11.05	**.001**	**.092**	**Medium**
	Fol	21	23	46.90	48.30	7.37	7.53	3.82	0.05	**.032**	**Small**
Italian Secondary school	Pre	19	17	41.47	38.76	8.46	7.00				
	Post	19	17	42.63	41.82	8.51	6.86	0.38	.540	.003	None
	Fol	19	17	40.11	40.65	9.42	7.42	1.06	.310	**.013**	**Small**

Note. Pre = pre-test; Post = post-test; Fol = follow-up; EG = intervention group; CG = control group. Significant results are shown in bold. Effect sizes are reported as partial eta squared (η^2^*p*).

**Table 4:** Interpersonal Competence (EQ-i:YV subscale).

Sample/ Subsample	Measure	N (EG)	N (CG)	Mean (EG)	Mean (CG)	SD (EG)	SD (CG)	F	*p*	η^2^*p*	Effect
Whole	Pre	82	84	37.60	37.44	5.16	5.54				
	Post	82	84	36.32	37.83	6.67	5.32	3.86	.050	**.010**	**Small**
	Fol	82	84	36.71	38.01	6.02	6.10	2.24	.140	.010	None
Spanish	Pre	42	44	37.98	34.25	5.21	4.46				
	Post	42	44	36.69	39.41	7.24	4.85	0.15	.700	.000	None
	Fol	42	44	36.31	38.86	6.61	5.91	0.05	.820	.000	None
Italian	Pre	40	40	37.20	34.35	5.13	4.96				
	Post	40	40	35.92	36.10	6.09	5.34	5.96	**.020**	**.040**	**Small**
	Fol	40	40	37.13	37.08	5.38	6.34	3.69	.060	**.030**	**Small**
Primary school	Pre	45	45	37.07	38.09	5.55	5.53				
	Post	45	45	35.96	38.36	7.03	5.30	1.17	.280	.010	None
	Fol	45	45	37.58	38.93	5.27	6.66	0.05	.820	.000	None
Secondary school	Pre	37	39	38.24	36.69	4.63	5.52				
	Post	37	39	36.76	37.23	6.27	5.35	3.36	.070	**.020**	**Small**
	Fol	37	39	35.65	36.95	6.75	5.38	5.12	**.030**	**0.04**	**Small**
Spanish Primary school	Pre	24	22	38.25	34.95	5.28	4.89				
	Post	24	22	37.17	39.23	7.28	5.62	0.18	.680	.000	None
	Fol	24	22	37.71	38.64	5.74	7.33	0.99	.320	**.010**	**Small**
Spanish Secondary school	Pre	18	22	37.61	39.55	5.26	3.97				
	Post	18	22	36.06	39.59	7.34	4.05	0.82	.370	**.013**	**Small**
	Fol	18	22	34.44	39.09	7.38	4.22	2.59	.120	**.038**	**Small**
Italian Primary school	Pre	21	23	35.71	35.35	5.67	4.72				
	Post	21	23	34.57	37.52	6.64	4.96	2.74	.110	**.040**	**Small**
	Fol	21	23	37.43	39.22	4.81	6.11	1.08	.300	**.020**	**Small**
Italian Secondary school	Pre	19	17	38.84	33.00	4.00	5.10				
	Post	19	17	37.42	34.18	5.18	5.38	3.89	.060	**.050**	**Small**
	Fol	19	17	36.79	34.18	6.07	5.57	2.83	.100	**.050**	**Small**

Note. Pre = pre-test; Post = post-test; Fol = follow-up; EG = intervention group; CG = control group. Significant results are shown in bold. Effect sizes are reported as partial eta squared (η^2^*p*).

**Table 5:** Intrapersonal Competence (EQ-i:YV subscale).

Sample/ Subsample	Measure	N (EG)	N (CG)	Mean (EG)	Mean (CG)	SD (EG)	SD (CG)	F	*p*	η^2^*p*	Effect
Whole	Pre	82	84	13.60	13.35	3.01	3.47				
	Post	82	84	13.01	13.74	3.93	4.14	2.42	.120	.009	None
	Fol	82	84	13.17	13.54	4.08	3.96	0.83	.360	.003	None
Spanish	Pre	42	44	14.07	14.09	4.04	3.63				
	Post	42	44	13.31	14.16	4.13	3.97	1.25	.270	.007	None
	Fol	42	44	13.29	13.61	4.20	4.02	0.11	.740	.003	None
Italian	Pre	40	40	13.10	12.52	3.76	3.14				
	Post	40	40	12.70	13.27	3.72	4.31	1.23	.270	**.011**	**Small**
	Fol	40	40	13.05	13.45	4.01	3.95	1.00	.320	.008	None
Primary school	Pre	45	45	13.20	13.38	3.50	3.67				
	Post	45	45	12.73	13.91	3.53	4.15	1.19	.280	.009	None
	Fol	45	45	12.69	13.67	3.84	4.00	0.65	.420	.005	None
Secondary school	Pre	37	39	14.08	13.31	4.36	3.28				
	Post	37	39	13.35	13.54	4.39	4.16	1.27	.260	.009	None
	Fol	37	39	13.76	13.38	4.34	3.96	0.19	.670	.000	None
Spanish Primary school	Pre	24	22	13.29	14.68	3.49	3.98				
	Post	24	22	12.79	14.59	3.50	3.61	0.15	.700	.002	None
	Fol	24	22	12.25	13.95	3.50	4.16	0.05	.820	.000	None
Spanish Secondary school	Pre	18	22	15.11	13.50	4.56	3.22				
	Post	18	22	14.00	13.73	4.87	4.34	1.54	.220	**.018**	**Small**
	Fol	18	22	14.67	13.27	4.73	3.94	0.03	.870	.000	None
Italian Primary school	Pre	21	23	13.10	12.13	3.59	2.91				
	Post	21	23	12.67	13.26	3.65	4.60	1.02	.320	**.018**	**Small**
	Fol	21	23	13.19	13.39	4.21	3.93	0.70	.410	**.012**	**Small**
Italian Secondary school	Pre	19	17	13.11	13.06	4.04	3.44				
	Post	19	17	12.74	13.29	3.90	4.03	0.19	.660	.003	None
	Fol	19	17	12.89	13.53	3.87	4.11	0.24	.630	.004	None

Note. Pre = pre-test; Post = post-test; Fol = follow-up; EG = intervention group; CG = control group. Significant results are shown in bold. Effect sizes are reported as partial eta squared (η^2^*p*).

**Table 6:** Adaptability (EQ-i:YV subscale).

Sample/ Subsample	Measure	N (EG)	N (CG)	Mean (EG)	Mean (CG)	SD (EG)	SD (CG)	F	*p*	η^2^*p*	Effect
Whole	Pre	82	84	27.50	27.58	6.69	6.52				
	Post	82	84	27.72	28.27	6.46	5.90	0.34	.560	.000	None
	Fol	82	84	27.78	28.65	6.79	6.26	0.75	.390	.002	None
Spanish	Pre	42	44	27.64	29.20	7.06	6.13				
	Post	42	44	27.76	29.23	7.21	5.62	0.01	.930	.000	None
	Fol	42	44	28.00	27.95	6.78	6.28	0.02	.900	.000	None
Italian	Pre	40	40	27.35	25.80	6.37	6.55				
	Post	40	40	27.67	27.23	5.65	6.08	0.83	.360	.005	None
	Fol	40	40	28.00	27.95	6.78	6.28	1.31	.260	.008	None
Primary school	Pre	45	45	28.47	29.13	6.50	6.11				
	Post	45	45	28.76	29.78	6.31	6.03	0.08	.780	.000	None
	Fol	45	45	29.24	30.91	6.99	5.96	0.61	.440	.003	None
Secondary school	Pre	37	39	26.32	25.79	6.82	6.60				
	Post	37	39	26.46	26.54	6.50	5.30	0.39	.540	.001	None
	Fol	37	39	26.00	26.05	6.18	5.60	0.20	.650	.001	None
Spanish Primary school	Pre	24	22	28.92	31.23	6.46	6.47				
	Post	24	22	29.71	31.59	6.94	5.80	0.07	.790	.000	None
	Fol	24	22	28.67	32.33	6.43	6.01	0.47	.496	.005	None
Spanish Secondary school	Pre	18	22	25.94	27.18	7.63	5.14				
	Post	18	22	25.17	26.86	6.90	4.40	0.11	.750	.001	None
	Fol	18	22	26.11	26.36	7.36	5.03	0.31	.590	.004	None
Italian Primary school	Pre	21	23	27.95	27.13	6.67	5.10				
	Post	21	23	27.67	28.04	5.46	5.85	0.38	.540	.005	None
	Fol	21	23	29.90	29.65	7.68	5.75	0.10	.754	.001	None
Italian Secondary school	Pre	19	17	26.68	24.00	6.14	7.92				
	Post	19	17	27.68	26.12	6.01	6.40	0.74	.400	.006	None
	Fol	19	17	25.89	25.65	5.01	6.40	1.65	.210	**.024**	**Small**

Note. Pre = pre-test; Post = post-test; Fol = follow-up; EG = intervention group; CG = control group. Significant results are shown in bold. Effect sizes are reported as partial eta squared (η^2^*p*).

## Data Availability

De-identified aggregate data analysed for this article are available from the corresponding author upon reasonable request. In this manuscript, data from the first author’s doctoral thesis, which was supervised by the other three co-authors, have been reanalysed. The thesis was published in Spanish under an Attribution-NonCommercial-NoDerivatives 4.0 International (CC BY-NC-ND 4.0) license (https://hdl.handle.net/11441/128897, accessed on 28 October 2025).

## Abbreviations

The following abbreviations are used in this manuscript:

EI	Emotional Intelligence
EQ-i:YV	Emotional Quotient Inventory: Youth Version
EQ	Emotional Quotient
GLM	General Linear Model
